# Association of high-density lipoprotein with P1NP and β-CTX in hospitalized patients with osteoporotic fractures: a retrospective cross-sectional study

**DOI:** 10.3389/fendo.2026.1736832

**Published:** 2026-02-11

**Authors:** Cheng-bai Zhu, Peng Zhou, Ke Lu, Chong Li, Yin-lin Wei, Jian Jin, Wen-bin Hu, Yi-jun Gao

**Affiliations:** 1 Department of Orthopedics, Affiliated Kunshan Hospital of Jiangsu University, Suzhou, Jiangsu, China; 2 Kunshan Municipal Health and Family Planning Information Center, Suzhou, Jiangsu, China; 3 Chronic Disease Department, Kunshan Center for Disease Control and Prevention, Suzhou, Jiangsu, China

**Keywords:** bone turnover biomarkers (BTMs), beta-C-terminal telopeptide of type I collagen (β-CTX), high-density lipoprotein (HDL), procollagen type I N-terminal propeptide (P1NP), osteoporotic fractures (OPFs)

## Abstract

**Background:**

The intricate relationship between bone turnover biomarkers (BTMs) and lipid profiles, particularly high-density lipoprotein (HDL), remains partially understood. This study aims to clarify how HDL is associated with BTMs, which are key indicators of bone resorption and formation, in patients hospitalized with osteoporotic fractures (OPFs). Understanding this relationship could offer new insights for osteoporosis treatment and influence future therapeutic strategies.

**Method:**

Conducted at the Affiliated Kunshan Hospital of Jiangsu University from January 2017 to August 2023, this retrospective cross-sectional study involved 4782 OPFs patients requiring hospitalization or surgery; after applying the exclusion criteria, the actual valid sample size used for analysis was 712 patients. The patient’s serum HDL levels were determined, followed by the assessment of the procollagen type I N-terminal propeptide (P1NP) and the beta-C-terminal telopeptide of type I collagen (β-CTX) as outcome variables. Adjustments were made for age, gender, body mass index (BMI), and other clinical variables. The association between HDL and P1NP and β-CTX was analyzed using generalized estimating equations (GEE). Nonlinear associations were assessed via generalized additive models (GAM), with stratified analyses and threshold assessments conducted for result validation.

**Results:**

A negative association was found between HDL levels and both β-CTX and P1NP. After adjusting for covariates, each unit increase in HDL corresponded to a decrease in P1NP by 14.37 (β = -14.37, 95% CI: -24.21 to -4.51, *P* < 0.01) and in β-CTX by 0.14 (β = -0.14, 95% CI: -0.22 to -0.06, *P* < 0.01). Threshold analysis revealed a linear relationship between P1NP and β-CTX.

**Conclusion:**

The findings reveal a negative correlation between HDL levels and both β-CTX and P1NP, suggesting a potential link between lipid metabolism and bone turnover. If validated in further studies, HDL could emerge as a predictive marker for BTMs, offering novel perspectives for osteoporosis management.

## 1 Introduction

Osteoporosis (OP) is a degenerative bone condition marked by decreased bone density and structural deterioration, resulting in weakened bones and a higher risk of fractures (1). Globally, the number of hospitalized patients with osteoporosis and diminished bone mass is rising each year. As the population ages, the incidence of osteoporotic fractures (OPFs) is also increasing (2, 3). Statistics indicate that approximately 9 million OPFs occur annually, averaging about 20 fractures per minute, and affecting an estimated 2 billion people globally (4). In China, a study in 2019 revealed that the prevalence of OPFs among individuals aged 50 and above was 6.46% for male and 29.13% for female, respectively. This rate escalates to 81% by the age of 80-89 (5). OPFs significantly impacts the physical and mental well-being of hospitalized patients, imposing a substantial economic and healthcare burden on society (6). It increases the risk of recurrent fractures, reduces the quality of life for hospitalized patients, and raises the likelihood of hospital admissions, disability, and death (7).

Recent research has revealed the key role of lipids in various metabolic disorders (8). Currently, there is increasing societal attention on diseases related to abnormal lipid metabolism and osteoporosis. Researchers have found that hospitalized patients with hyperlipidemia may experience bone loss and osteoporosis, ultimately leading to fractures (9). Interestingly, higher levels of high-density lipoprotein (HDL) in individuals (≥135 mg/dl for women and ≥116 mg/dl for men) have been linked to an increased risk of all-cause mortality. Additionally, research has shown a positive association between HDL concentrations and increased death rates (10). These results have sparked a reassessment of HDL’s function in metabolic processes. The connection between HDL and bone metabolism has garnered significant attention. Studies have recognized increased HDL levels as a standalone risk factor for bone loss in both genders (11). Exploring HDL’s impact on bone metabolism may offer new theoretical insights for preventing and managing bone loss and enhance our understanding of osteoporosis. This research holds significant clinical relevance.

Timely screening, diagnosis, and intervention are vital for managing OPFs. They play a key role in lowering fracture risk, boosting patient quality of life, and mitigating the economic impact on society (12, 13). Bone density measurement can be utilized as a method for early OPFs diagnosis. However, the application of bone density measurement in early diagnosis is relatively limited (14). Hence, clinical research should consider alternative indicators for predicting and diagnosing OPFs. Bone turnover biomarkers (BTMs) are a series of biomarkers released during the bone remodeling process that can detect short-term changes in bone turnover, which are linked to a higher risk of fractures (15). They can be assessed in blood, plasma, or urine. In this study, procollagen type I N-terminal propeptide (P1NP) and beta-C-terminal telopeptide of type I collagen (β-CTX) have been recommended as reference BTMs for predicting fracture risks and monitoring osteoporosis (14, 16). P1NP is a crucial component of bone matrix that reflects bone cell formation. As a BTMs, it is commonly used to assess OPFs or estimate the prognosis of patients with bone metabolic diseases (17, 18). β-CTX is a peptide segment resulting from collagen degradation that can be evaluated through immunological analysis using conformation antibodies, which specifically recognize the bone resorption process and serve as an important metabolic indicator. β-CTX and P1NP are frequently employed to evaluate OPFs and forecast the outcomes for individuals with bone metabolic disorders (19, 20).

Previous research indicates a potential association between HDL levels and the internal microenvironment (21). This internal microenvironment can influence the differentiation and function of osteoblasts, potentially leading to bone loss (21). However, the association between HDL and OPFs, particularly its relationship with bone turnover markers, has not been thoroughly explored due to limited available data. Hence, this study aims to explore the association between HDL concentrations and β-CTX and P1NP in patients hospitalized for OPFs, with the goal of filling this research gap (22).

## 2 Materials and methods

### 2.1 Ethical statement

The study received approval from the Ethics Committee of the Affiliated Kunshan Hospital of Jiangsu University, Suzhou, China (approval No. 2021–06-015-K01), and adhered to the principles outlined in the Declaration of Helsinki. The hospitalized patients’ identities were concealed to ensure an unbiased investigation. All hospitalized patients provided written informed consent.

### 2.2 Research framework and clinical participant groups

The research involved a retrospective cross-sectional evaluation of data from hospitalized patients with acute OPFs patients collected between January 2017 and August 2023. The medical records of these hospitalized patients were obtained from Affiliated Kunshan Hospital of Jiangsu University. A total of 4782 hospitalized with acute OPFs patients who received operative intervention were included in the study. During their hospital stay, each participant had blood samples taken for testing. The diagnostic criteria for OP included: (1) the existence of bone instability, bone loss, and fractures, alongside reduced standard bone mineral density (BMD) (T-score) as indicators of osteoporosis; bone density measurements, performed during the hospitalization of fracture patients, were included as part of this assessment; (2) the OP diagnostic criteria set by the World Health Organization, which state a T-score ≤ -2.5, even in the absence of widespread fractures (23).

The inclusion criteria for OPFs hospitalized patients are as follows: (1) individuals aged 50 years and above; (2) possessing complete demographic information; (3) undergoing lipid assessment and bone turnover marker testing. The criteria for exclusion include: (1) incomplete or missing documentation; (2) any history of cancer; (3) metabolic conditions related to the liver or kidneys; (4) aside from diabetes, other endocrine-related disorders, such as polycystic ovary syndrome and Cushing’s syndrome, should be considered regardless of their temporal presentation (past or current). (5) use of medications for osteoporosis prevention or lipid regulation; (6) multiple or pathological hip fractures (24).

**Figure 1:**
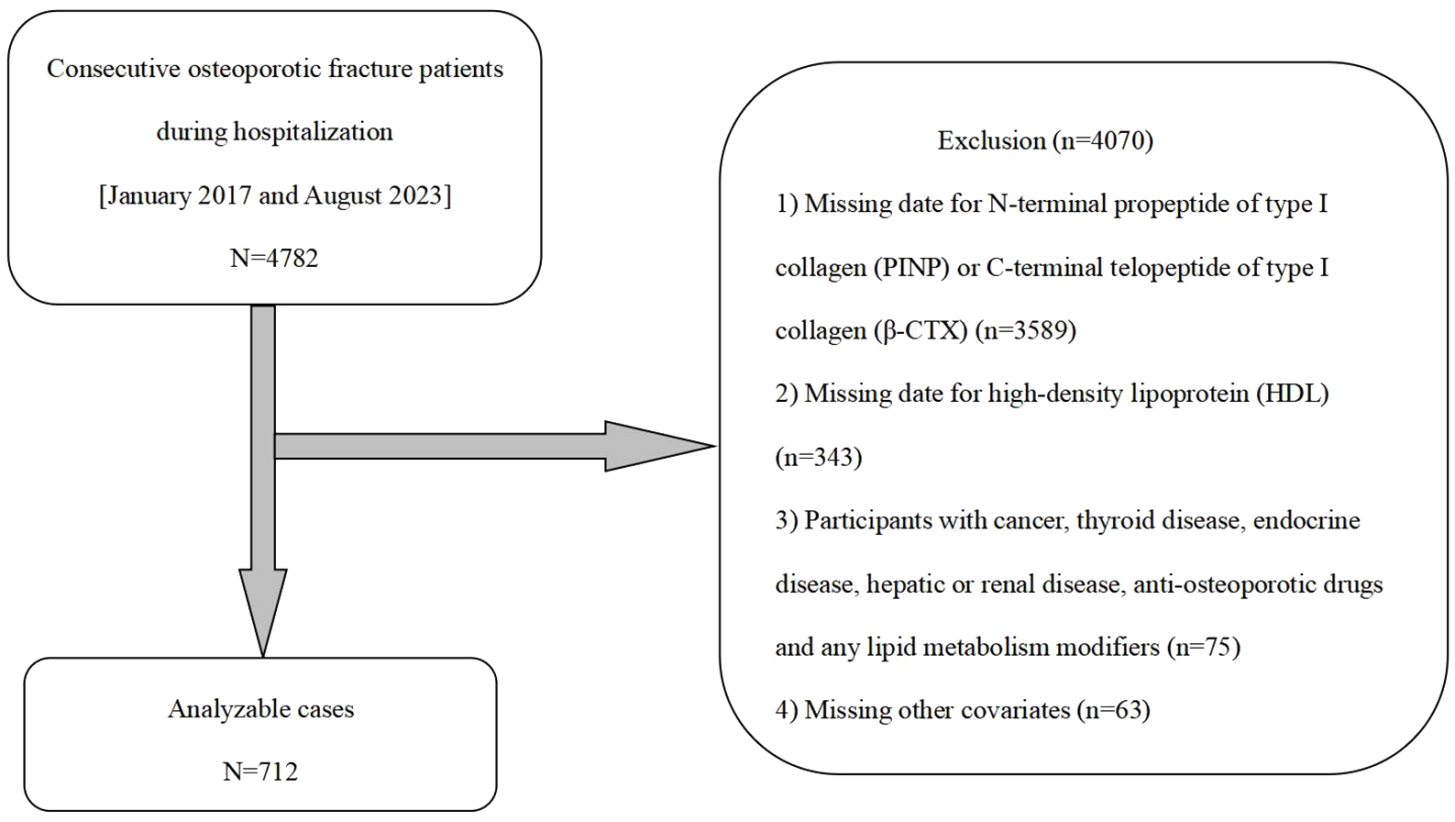
Research workflow diagram.

Initially, this research project involved a total of 4782 hospitalized OPFs patients. However, 3589 OPFs hospitalized patients were excluded due to the lack of data on P1NP and β-CTX, and an additional 343 OPFs hospitalized patients were excluded due to the lack of HDL data. Furthermore, 75 hospitalized patients were excluded from the research based on the following eligibility criteria: history of cancer, thyroid disorders, endocrine diseases, schistosomiasis-associated nephropathy, use of anti-osteoporosis medications, and any lipid-modulating agents (24). And another 63 OPFs hospitalized patients had missing other covariates. Therefore, the final study cohort comprised 712 OPFs hospitalized patients. The screening process is illustrated in **Figure 1**.

### 2.3 Methods of research

To guarantee the reliability and impartiality of the data, all researchers completed standardized training prior to the study’s initiation. They utilized a standardized survey approach to gather essential information from OPFs hospitalized patients. This included comprehensive medical histories covering various malignants, liver and renal diseases, thyroid and endocrine disorders, as well as the use of osteoporosis medications and lipid metabolism modifiers. After the survey was completed, the data were compiled, summarized, and verified. Following this, researchers measured participants’ height, body weight, and blood pressure in the morning, after a 12-hour fasting period. Each participant was measured twice, and the average of these measurements was used to minimize potential inaccuracies. BMI is calculated as weight divided by the square of height (kg/m²) (24).

### 2.4 Measurements in the laboratory

All measurements were performed on the morning of the third day prior to hospital admission after an overnight fast. The following laboratory parameters were determined: HDL, alanine aminotransferase (ALT), aspartate aminotransferase (AST), fasting blood glucose (FBG), serum calcium, serum creatinine (CR), uric acid (UA), and urea nitrogen (UN). Levels of BTMs, including P1NP and β-CTX, were assessed in the hospitalized patients. The Roche Diagnostics (Mannheim, Germany) ECLIA approach was used to quantify the concentrations of β-CTX and P1NP. All measurements were performed by experienced personnel using the same instrumentation and standardized protocols.

### 2.5 Variables

Drawing on prior research, clinical protocols, our database, and preliminary work from our team (24–26). In this research, we considered β-CTX and P1NP as dependent variables, while HDL was treated as the independent variable. A variety of covariates were considered, including age, gender, BMI, ALT, AST, serum CR, UA, UN, serum calcium, the Charlson comorbidity index (CCI) (27), FBG, alcohol consumption, smoking status, hypertension, and diabetes. The hospitalized patients’ physical condition and comorbidities were evaluated by the Charlson Comorbidity Index (CCI). Regular alcohol use was identified as consuming alcohol weekly over the last 12 months, Tobacco use was classified as being either a active or previous smoker within the same period (28). Fracture categories included fractures of the wrist, thoracic vertebra, lumbar vertebra, and femoral trochanteric region.

### 2.6 Statistical analyses

Normality tests were performed on all data, and sociodemographic, laboratory, and clinical data were expressed as median (25^th^ and 75^th^ percentile) ± standard deviation (SD). Categorical data reported as counts and percentages. Univariate analysis of categorical data utilized Fisher’s exact or Pearson’s chi-square tests. For continuous data with non-normal and normal distributions, the Mann-Whitney U test and t-test were used, respectively. Single-variable analyses were conducted to examine the link between OPFs patient attributes and P1NP and β-CTX levels. A multiple linear regression analysis was used to investigate the relationship between HDL concentrations and BTMs in hospitalized OPFs patients.

### 2.7 Model construction

Generalized Estimating Equations (GEE) and Generalized Additive Models (GAM) are two fundamental models in statistics. GEE seeks to evaluate the overall average effect by specifying a working correlation structure suitable for managing correlated data, such as longitudinal or clustered data, and is applicable to response variables that do not follow a normal distribution. In contrast, GAM employs flexible non-parametric smoothing functions to examine complex nonlinear relationships between response and predictor variables without dependence on parametric forms. Both models require the specification of the response distribution, the development of an average model, and the utilization of iterative algorithms for parameter estimation. Researchers can assess model fit and perform statistical inference based on these models.

GEE was applied in this study to uncover the independent connections between HDL levels, P1NP, and β-CTX in OPFs hospitalized patients, while accounting for potential confounding factors. Three models were created: the basic model (Model I), the partially adjusted model (Model II), and the comprehensively adjusted model (Model III). Initially, the variance inflation factor (VIF) was used to evaluate multicollinearity among the covariates. Subsequently, variables were adjusted with the following criteria: (1) a substantial shift of at least 10% in the odds ratio (OR) following the addition or removal of covariates in the initial or comprehensive model; (2) covariates that either satisfy Standard 1 or show a *p*-value less than 0.1 in Model I Model II and III employed Standard 1 and Standard 2 for covariate adjustments. Specifically, Model II (the minimally adjusted model) considered age, gender, BMI, ALT, and AST; whereas Model III considered age, gender, BMI, ALT, AST, UA, UN, serum CR, serum calcium, FBG, diabetes, hypertension, alcohol consumption, smoking status, and fracture category.

Using a GAM to assess potential nonlinear associations, employing a two-piece linear regression model to generate smooth curves and identify threshold effects. Employing recursive techniques based on the maximum likelihood model to determine inflection points of these curves when significant proportions are observable (29). After stratification, the robustness and subgroup variability of the study patients are assessed based on specific variables. Likelihood ratio tests (LRT) are conducted using subgroup interactions and modifiers.

All the statistical evaluations were performed using the R Foundation packages1 and Empower Stats from X&Y Solutions, 1 www.R-project.org Inc., MA, USA. *P* < 0.05 was deemed as the significance level fora two-sided test.

## 3 Result

### 3.1 Features of hospitalized patients

Referring to the inclusion criteria outlined in **Figure 1**, 712 OPFs hospitalized patients who received treatment between January 2017 and August 2023 were included for analysis. **Table 1** provides an overview of the foundational traits of the hospitalized patient group (n=712), consisting of 71.21% women and 28.79% men. The mean age was 68.80 ± 10.84. The overall mean values for HDL, β-CTX, and P1NP were 1.33 ± 0.29, 0.55 ± 0.30, and 58.05 ± 36.67, respectively. The table demonstrates notable variations in specific markers, including AST, serum calcium, serum CR, UA, P1NP, β-CTX, and HDL, between the groups. However, factors such as age, BMI, ALT, UN, FBG, and CCI exhibited no significant differences. Understanding the characteristics of different groups and their potential impact on the study outcomes is crucial based on this information.

**Table 1:** Characteristics of study participants.

HDL, mmol/L	Total (1.04 - 1.62)	Low (0.55 - 1.29)	High (1.30 - 2.44)	*P*-value	*P*-value^*^
	Mean ± SD	Mean ± SD	Mean ± SD		
Age, years	68.80 ± 10.84	69.46± 10.86	68.17 ± 10.79	0.11	0.09
BMI, kg/m^2^	23.05 ± 3.26	22.96 ± 3.16	23.13 ± 3.34	0.47	0.36
ALT, U/L	22.01 ± 15.53	22.15 ± 13.50	21.87 ± 17.25	0.81	0.56
AST, U/L	24.56 ± 14.19	23.84 ± 11.83	25.23 ± 16.10	0.19	0.01
Serum calcium, mmol/L	2.22 ± 0.12	2.21 ± 0.12	2.23 ± 0.13	0.04	0.04
Serum CR, µmoI/L	63.00 ± 22.58	67.09 ± 26.05	59.14 ± 17.90	<0.01	<0.01
UA, µmoI/L	92 ± 88.69	293.26 ± 93.46	261.46 ± 81.08	<0.01	<0.01
UN, mmol/L	6.03 ± 2.03	6.15 ± 2.26	5.92 ± 1.78	0.13	0.55
FBG, mmol/L	6.01 ± 1.83	6.08 ± 1.94	5.93 ± 1.73	0.27	0.35
P1NP, µg/L	58.05 ± 36.67	61.63 ± 44.78	54.67 ± 26.46	0.01	0.01
β-CTX, ng/mL	0.55 ± 0.30	0.58 ± 0.31	0.53 ± 0.28	0.01	0.01
CCI, N (%)					
0	649 (91.15%)	316 (91.33%)	333 (90.98%)		0.87
≥1	63 (8.85%)	30 (8.67%)	33 (9.02%)		
Gender, N (%)					0.27
Female	507 (71.21%)	253 (73.12%)	254 (69.40%)		
Male	205 (28.79%)	93 (26.88%)	112 (30.60%)		
Alcohol consumption, N (%)					0.32
No	684 (96.07%)	335 (96.82%)	349 (95.36%)		
Yes	28 (3.93%)	11 (3.18%)	17 (4.64%)		
Smoking status, N (%)					0.17
No	668 (93.82%)	329 (95.09%)	339 (92.62%)		
Yes	44 (6.18%)	17 (4.91%)	27 (7.38%)		
Hypertension, N (%)					0.33
No	628 (88.20%)	301 (86.99%)	327 (89.34%)		
Yes	84 (11.80%)	45 (13.01%)	39 (10.66%)		
Diabetes, N (%)					0.95
No	687 (96.49%)	334 (96.53%)	353 (96.45%)		
Yes	25(3.51%)	12 (3.47%)	13 (3.55%)		
Fracture category, N (%)					0.41
Thoracic vertebra	125 (17.56%)	61 (17.63%)	64 (17.49%)		
Lumbar vertebra	221 (31.04%)	98 (28.32%)	123 (33.61%)		
Wrist	39 (5.48%)	18 (5.20%)	21 (5.74%)		
Proximal humerus	87 (12.22%)	49 (14.16%)	38 (10.37%)		
Femoral trochanteric	240 (33.70%)	120 (34.69%)	120 (32.79%)		

*Kruskal-Wallis rank test for continuous variables, Fisher exact for categorical variables with expects < 10.

SD, standard deviation; CI, confidence interval; BMI, body mass index; ALT, alanine aminotransferase; AST, aspartate aminotransferase; Serum CR, serum creatinine; UA, uric acid; UN, urea nitrogen; FBG, fasting blood glucose; P1NP, procollagen type I N-terminal propeptide; β-CTX, beta-C-terminal telopeptide of type I collagen; HDL, high-density lipoprotein; CCI, Charlson comorbidity index.

### 3.2 Univariate analysis of bone turnover biomarkers

**Table 2** presents the outcomes of the single-variable analysis for various factors associated with P1NP and β-CTX. For P1NP, FBG levels and hypertension are significantly associated with its levels. Higher FBG levels are significantly negatively correlated with lower P1NP levels (β = -2.82, 95% CI: -4.27 to -1.36, *P* < 0.01) (β, as a coefficient in the linear regression equation, indicating both the strength and direction of the relationship between HDL and BTMs), while hypertension is significantly positively correlated with P1NP levels (β = 10.17, 95% CI: 18.49 to 1.85, *P* = 0.02). Other variables such as age, BMI, ALT, and AST do not significantly impact P1NP. For β-CTX, FBG and UA levels exert significant effects. FBG is inversely associated with β-CTX levels (β = -0.03, 95% CI: -0.04 to -0.01, *P* < 0.01), and UA levels also show an inverse relationship with β-CTX (β = -0.0003, 95% CI: -0.0006 to -0.0001, *P* = 0.01). Additionally, both ALT (β = -0.0020, 95% CI: -0.0034 to -0.0007, *P* < 0.01) and AST (β = -0.0024, 95% CI: -0.0039 to -0.0007, *P* < 0.01) exhibit a negative correlation with β-CTX levels. Factors like gender, BMI, serum calcium, serum CR, alcohol consumption, and smoking do not significantly affect β-CTX.

**Table 2:** Univariate analysis for BTMs.

HDL, mmol/L	Statistics^a^	P1NP β^b^(95%CI) *p*-value	β-CTX β^b^(95%CI) *p*-value
Age, years	68.79 ± 10.84	0.06 (-0.19, 0.31) 0.62	**0.0001 (-0.0019, 0.0021)** 0.89
BMI, kg/m^2^	23.05 ± 3.30	0.04 (-0.79, 0.86) 0.93	**0.0038 (-0.0029, 0.0104)** 0.27
ALT, U/L	22.01 ± 15.53	-0.11 (-0.29, 0.06) 0.20	**-0.0020 (-0.0034, -0.0007)** <0.01
AST, U/L	24.55 ± 14.19	**-0.0009 (-0.19, 0.19)** **0.99**	**-0.0024 (-0.0039, -0.0007)** <0.01
Serum calcium, mmol/L	2.22 ± 0.12	8.04 (-13.77, 29.85) 0.47	0.14 (-0.03, 0.32) 0.12
Serum CR, µmoI/L	63.00 ± 22.58	0.08 (-0.04, 0.19) 0.22	**0.0010 (-0.0019, 0.0001)** 0.05
FBG, mmol/L	6.01 ± 1.83	-2.82 (-4.27, -1.36) <0.01	-0.03 (-0.04, -0.01) <0.01
UA, µmoI/L	276.92 ± 88.69	-0.02 (-0.05, 0.01) 0.31	**-0.0003 (-0.0006, -0.0001)** 0.01
UN, mmol/L	6.03 ± 2.03	0.14 (-1.19, 1.47) 0.83	**-0.0010 (-0.0117, 0.0097)** 0.85
Gender, N (%)			
Female	507 (71.21%)	Reference	Reference
Male	205 (28.79%)	0.96 (-5.01, 6.90) 0.76	-0.01 (-0.06, 0.03) 0.59
CCI, N (%)			
0	649 (91.15%)	Reference	Reference
≥1	63 (8.85%)	7.40 (-2.07, 16.88) 0.13	-0.02 (-0.10, 0.05) 0.57
Alcohol consumption, N (%)			
No	684 (96.07%)	Reference	Reference
Yes	28 (3.93%)	-4.54 (-18.40, 9.32) 0.52	-0.04 (-0.15, 0.07) 0.51
Smoking status, N (%)			
No	668 (93.82%)	Reference	Reference
Yes	44 (6.18%)	-1.40 (-12.60, 9.79) 0.81	-0.06 (-0.15, 0.03) 0.21
Hypertension, N (%)			
No	628 (88.20%)	Reference	Reference
Yes	84 (11.80%)	10.17 (1.85, 18.49) 0.02	0.03 (-0.04, 0.10) 0.39
Diabetes, N (%)			
No	687 (96.49%)	Reference	Reference
Yes	25 (3.51%)	-1.61 (-16.26,13.03) 0.83	-0.04 (-0.15, 0.08) 0.54
Fracture category, N (%)			
Thoracic vertebra	125 (17.56%)	Reference	Reference
Lumbar vertebra	221 (31.04%)	-3.16 (-11.20, 4.88) 0.44	-0.04 (-0.11, 0.02) 0.19
Wrist	39 (5.48%)	-14.25 (-27.43, -1.8) 0.03	-0.18 (-0.28, -0.07) <0.01
Proximal humerus	87 (12.22%)	-4.27 (-14.30, 5.76) 0.41	-0.07 (-0.15, 0.01) 0.07
Femoral trochanteric	240 (33.70%)	-2.76 (-10.68, 5.16) 0.50	-0.08 (-0.14, -0.02) 0.01

Bolded data are now presented with four decimal places, which improves the sensitivity of the data.

a For continuous variables.

b The dependent variable was bone turnover markers and β is the result of univariate analysis for bone turnover markers.

CI, confidence interval; BMI, body mass index; ALT, alanine aminotransferase; AST, aspartate aminotransferase; Serum CR, serum creatinine; UA, uric acid; UN, urea nitrogen; FBG, fasting blood glucose; P1NP, procollagen type I N-terminal propeptide; β-CTX, beta-C-terminal telopeptide of type I collagen; BTMs, bone turnover markers; CCI, Charlson comorbidity index.

### 3.3 Analysis of the relationship between HDL and bone turnover biomarkers

**Table 3** presents the analysis results of the association between HDL levels and P1NP in different models. In the unadjusted model I, HDL levels were significantly negatively associated with P1NP levels (β = -10.19, 95% CI: -19.56 to -0.82, *P* = 0.03). This inverse relationship continued to be significant in Model II, even after accounting for age, gender, BMI, ALT, and AST (β = -11.23, 95% CI: -20.68 to -1.78, *P* = 0.02). In the extended Model III, additional adjustments for serum calcium, serum CR, UA, UN, CCI, FBG, alcohol use, smoking habits, hypertension, diabetes, and fracture category were made, revealing an even stronger negative association (β = -13.80, 95% CI: -23.67 to -3.93, *P* < 0.01). These findings highlight a substantial relationship between higher HDL levels and lower P1NP levels, with the effect becoming more pronounced after accounting for various influencing factors.

**Table 3:** Association between HDL and P1NP in different models.

HDL, mmol/L	Model I^a^ N=712		Model II^b^ N=712		Model III^c^ N=712	
	β(95%CI)	*P* value	β(95%CI)	*P* value	β(95%CI)	*P* value
HDL, mmol/L	-10.19 (-19.56, -0.82)	0.03	-11.23 (-20.68, -1.78)	0.02	-13.80 (-23.67, -3.93)	<0.01
Dichotomous HDL, mmol/L						
Low (0.55 - 1.29)	Reference		Reference		Reference	
High (1.30 - 2.44)	-12.92 (-28.11, 2.28)	0.10	-13.41 (-28.69, 1.87)	0.09	-12.37 (-27.98, 3.24)	0.12

a adjust for: none.

b adjust for age, gender, BMI, ALT, AST.

c adjust for age, gender, BMI, ALT, AST, Serum calcium, Serum CR, UA, UN, CCI, FBG, alcohol consumption, smoking status, hypertension, diabetes, fracture category.

CI, confidence interval; BMI, body mass index; ALT, alanine aminotransferase; AST, aspartate aminotransferase; Serum CR, serum creatinine; UA, uric acid; UN, urea nitrogen; FBG, fasting blood glucose; P1NP, procollagen type I N-terminal propeptide; HDL, high-density lipoprotein.

**Table 4** demonstrates the connection between HDL levels and β-CTX. In Model I, a 1 mmol/L increase in HDL was linked to a notable reduction in β-CTX levels (β = -0.10, 95% CI: -0.18 to -0.03, *P* = 0.01), signifying a negative relationship between HDL and β-CTX. After controlling for age, gender, BMI, ALT, and AST, the association persisted in Model II (β = -0.09, 95% CI: -0.17 to -0.02, *P* = 0.01).Additional adjustments for factors such as serum calcium, serum CR, and UA in Model III showed that each 1 mmol/L rise in HDL was significantly associated with a reduction in β-CTX levels (β = -0.13, 95% CI: -0.21 to -0.05, *P* < 0.01), underscoring the continued presence of this negative relationship after accounting for various confounding variables. Moreover, the high HDL group displayed notably lower β-CTX levels compared to the low HDL group. This finding remained significantly consistent across all models, reinforcing the negative relationship between HDL and markers of bone metabolism.

**Table 4:** Association between HDL and β-CTX in different models.

HDL, mmol/L	Model I^a^ N=712		Model II^b^ N=712		Model III^c^ N=712	
	β (95%CI)	*P* value	β (95%CI)	*P* value	β (95%CI)	*P* value
HDL, mmol/L	-0.10 (-0.18, -0.03)	0.01	-0.09 (-0.17, -0.02)	0.01	-0.13 (-0.21, -0.05)	<0.01
Dichotomous HDL, mmol/L						
Low (0.55 - 1.29)	Reference		Reference		Reference	
High (1.30 - 2.44)	-0.06 (-0.10, -0.01)	0.01	-0.06 (-0.10, -0.01)	0.01	-0.07 (-0.11, -0.03)	<0.01

a adjust for: none.

b adjust for age, gender, BMI, ALT, AST.

c adjust for age, gender, BMI, ALT, AST, Serum calcium, Serum CR, UA, UN, CCI, FBG, alcohol consumption, smoking status, hypertension, diabetes, fracture category.

CI, confidence interval; BMI, body mass index; ALT, alanine aminotransferase; AST, aspartate aminotransferase; Serum CR, serum creatinine; UA, uric acid; CCI, Charlson comorbidity index; UN, urea nitrogen; FBG, fasting blood glucose; β-CTX, beta-C-terminal telopeptide of type I collagen; HDL, high-density lipoprotein.

### 3.4 Stratified analysis in subgroups

**Figure 2:**
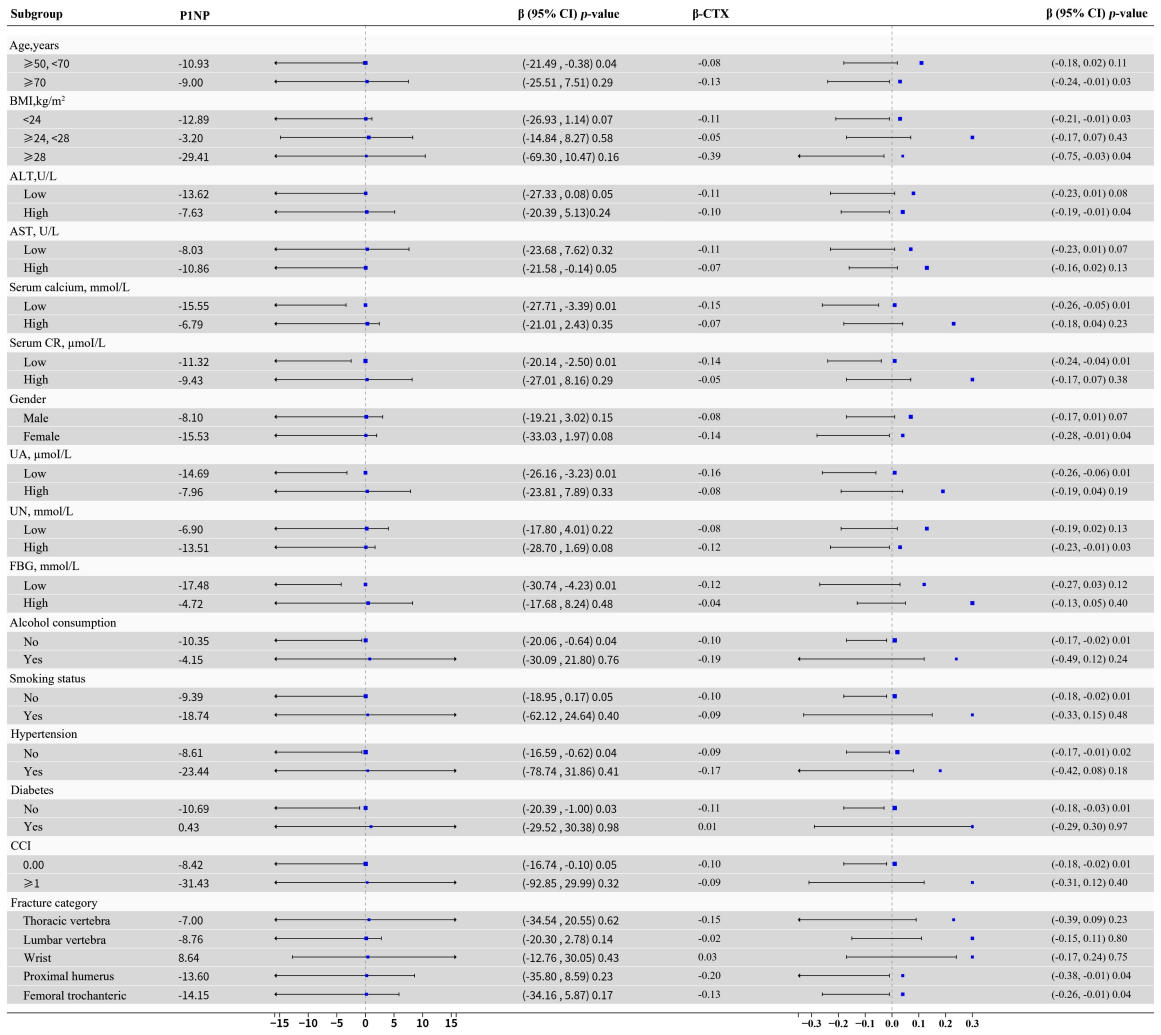
Patients were categorized according to age, gender, BMI, ALT, AST, serum calcium, serum CR, UA, UN, CCI, FBG, alcohol consumption, smoking status, hypertension, diabetes, fracture category. Other variables not accounted for in the stratification were controlled for in the analysis. CI, confidence interval; BMI, body mass index; ALT, alanine aminotransferase; AST, aspartate aminotransferase; serum CR, serum creatinine; UA, uric acid; CCI, Charlson comorbidity index; UN, urea nitrogen; FBG, fasting blood glucose; P1NP, procollagen type I N-terminal propeptide; β-CTX, beta-C-terminal telopeptide of type I collagen; HDL, high-density lipoprotein; BTMs, bone turnover markers.

Classify OPFs patients according to factors such as age, gender, BMI, smoking status, alcohol intake CCI, hypertension, diabetes, FBG, ALT, AST, serum calcium, and serum CR. Further perform subgroup analysis to evaluate the robustness of Model 3. Adjust for covariates not used in stratification. The findings reveal a uniform trend, with no evidence of interactions observed as a result of stratification (all *P* > 0.05, **Figure 2**).

### 3.5 Spline smoothing graph and threshold exploration

**Figure 3:**
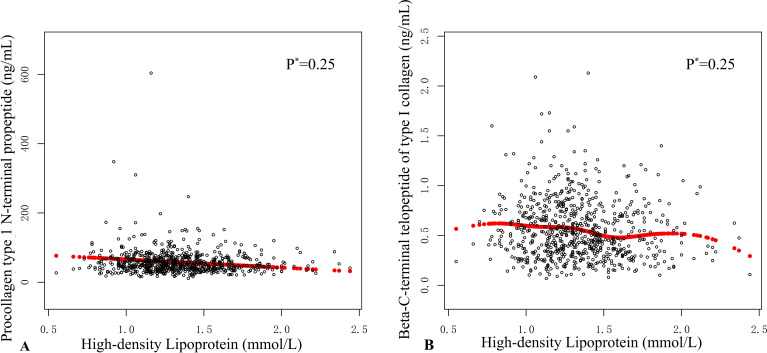
Smooth curve analysis was employed to elucidate the relationship between high-density lipoprotein (HDL) levels and the measurements of procollagen type I N-terminal propeptide (P1NP) and the beta-C-terminal telopeptide of type I collagen (β-CTX). In **(A, B)**, each black dot represents an individual participant's sample. **(A)** illustrates the relationship between HDL levels and P1NP, while **(B)** displays the relationship between HDL levels and β-CTX. The red curve indicates the overall trend of the sample data, revealing a potential association between HDL levels and both P1NP and β-CTX. *The p-values were calculated using the likelihood ratio test (LRT).

The association among HDL, β-CTX, and P1NP was assessed through graphical methods to identify whether the relationship was linear or nonlinear. In the **Figure 3**, each black dot represents an individual participant’s data, illustrating their measurements for HDL, P1NP, and β-CTX. The graphical display shows a linear effect. By conducting threshold analysis, we investigated the relationship between HDL levels and the BTMs β-CTX and P1NP in a cohort of hospitalized OPFs patients to ascertain whether the relationship is linear or nonlinear (**Table 5**). GAM estimation revealed that, after accounting for covariates, a linear relationship exists between HDL and BTMs in the OPFs group. Threshold analysis of HDL and BTMs further indicated that there is no inflection point.

**Table 5:** Threshold analyses examining the relationship between HDL and BTMs.

HDL, mmol/L	Model III^a^	
	P1NP β (95% CI) *P*-value	β-CTX β (95% CI) *P*-value
Model A^b^		
One line slope	-13.81 (-23.70, -3.93) 0.01	-0.13 (-0.21, -0.05) <0.01
Model B^c^		
BTMs turning point (K)	1.55	1.56
<K	-19.77 (-34.08, -5.46) 0.01	-0.18 (-0.29, -0.07) <0.01
>K	0,03 (-25.95, 26.02) 0.99	-0.02 (-0.23, 0.19) 0.85
Slope 2-Slope 1	19.80 (-14.59, 54.20) 0.26	0.16 (-0.11, 0.43) 0.26
LRT^d^	0.25	0.25

a Adjusted for age, gender, BMI, ALT, AST, Serum calcium, Serum CR, UA, UN, CCI, FBG, alcohol consumption, smoking status, hypertension, diabetes, fracture category.

b Linear analysis, p value<0.05 indicates a linear relationship.

c Nonlinear analysis.

d *P*-value <0.05 means Model B is not significantly different from Model A, which indicates a linear relationship.

BMI, body mass index; ALT, alanine aminotransferase; AST, aspartate aminotransferase; Serum CR, serum creatinine; UA, uric acid; UN, urea nitrogen; FBG, fasting blood glucose; HDL, high-density lipoprotein; P1NP, procollagen type I N-terminal propeptide; CCI, Charlson comorbidity index; β-CTX, beta-C-terminal telopeptide of type I collagen; SIRI, systemic inflammatory response index; BTMs, bone turnover markers.

## 4 Discussion

OPFs presents a growing public health concern within the framework of China’s aging population (30–32). The occurrence and intensity of this condition increase markedly with an aging population. OPFs emerge as common contributors to illness and death among the elderly (33). This cross-sectional study seeks to explore the association between HDL levels and BTMs, such as β-CTX and P1NP, in 712 hospitalized cases of osteoporotic fractures. The research results suggest that higher HDL levels are associated with decreased bone turnover marker levels, implying that lower HDL levels might offer a protective benefit to the skeletal system.

The concentrations of estrogen and testosterone, which are essential in controlling bone metabolism, have been linked to variations in HDL levels (34). HDL levels are also associated with the distribution and metabolism of adipose tissue. Fat tissue releases several substances that affect bone metabolism, suggesting that HDL might influence bone density by altering lipid metabolism (35). HDL is a type of lipid known for its anti-atherogenic effects and possible anti-inflammatory properties. Bone cell activity can be inhibited by inflammatory factors, meaning that the anti-inflammatory properties of HDL could play a role in supporting bone density (36). Due to HDL’s complex role in bone density, researchers are currently investigating novel markers to more accurately evaluate HDL’s effect on skeletal turnover.

Previous clinical studies have primarily focused on the relationship between HDL and BMD. Some research points to an inverse relationship between HDL and BMD, whereas other findings reveal a positive connection (34). Although the link between HDL and BMD has received increasing interest in recent years, the precise nature of this relationship remains uncertain (33, 37). Recently, there has been growing interest in using BTMs as an alternative measure to evaluate and manage BMD in hospitalized osteoporosis patients (38–40). BTMs levels can provide insight into the overall skeletal metabolic condition, with some indications that in particular clinical contexts, BTMs might better account for variations in bone quality (41, 42). Therefore, this study regards BTMs as the dependent variable in osteoporotic fracture hospitalized patients, modulating the bone conversion process and reflecting the dynamic state of bone metabolism. At present, the interaction between lipid metabolism and bone health has generated considerable interest among researchers (43, 44). Rising biological and clinical epidemiological data underscores the relationship between fractures and osteoporosis, with lipid metabolism functioning as a bridge between these two issues. Individuals with hyperlipidemia may simultaneously experience bone loss and osteoporotic fractures. Significantly, patients with osteoporotic fractures and diminished bone density who are hospitalized frequently show disturbances in lipid metabolism, especially in HDL metabolism (39).

The current study highlights the association between HDL and P1NP and β-CTX. Upon adjusting for relevant variables, a negative association was observed between HDL and P1NP (β = -14.37; 95% CI: -24.21 to -4.51; *P* < 0.01). The threshold analysis showed a highly linear trend between HDL and P1NP (**Table 5**). P1NP acts as a marker for evaluating the factors affecting changes in bone mineral density. P1NP known for its high specificity as a BTMs (45). Moreover, β-CTX, reflects the activity of osteoclasts (46). β-CTX serves as another indicator of bone metabolism. The threshold analysis revealed a highly linear relationship between HDL and β-CTX (**Table 5**). Clinical studies indicate that an elevation in serum β-CTX concentration is associated with increased bone resorption, heightened fracture risk, and accelerated bone mineral loss (47). Clinical research shows that higher serum β-CTX levels are linked to greater bone resorption, an increased risk of fractures, and more rapid loss of bone mineral (47). The International Federation of Clinical Chemistry and Laboratory Medicine (IFCC) and the International Osteoporosis Foundation (IOF) suggest that serum concentrations of P1NP and β-CTX can serve as reference bone turnover markers (48). In this investigation, we detected a significant inverse linear correlation between HDL concentrations and β-CTX (β = -0.14; 95% CI: -0.22 to -0.06; *P* < 0.01). These findings suggest that β-CTX decreases with increasing HDL levels, highlighting a decrease in bone resorption. This validates the conclusion that elevated HDL levels could lead to a reduction in bone turnover markers, indicating a potential mechanism for decreased bone metabolism.

HDL is essential for modulating the development and activation of osteoblasts and osteoclasts, which in turn affects bone integrity (34). HDL levels are negatively associated with BTMs, meaning that as HDL levels increase, BTMs decrease This could be attributed to HDL’s detrimental impact on bone metabolism, potentially by affecting osteoblast development and increasing the number of bone marrow adipocytes (49). It is a key lipid in the human body, associated with various metabolic diseases. Earlier research points to a possible association between HDL and osteoporosis fractures in hospitalized patients, suggesting that HDL and bone metabolism may be connected through hormonal imbalances and inflammatory processes (39). In hospitalized patients with OPFs, especially obese males, the reduction in BTMs with high HDL is particularly pronounced. This might be linked to irregularities in male HDL metabolism and the negative impact of HDL on bone health associated with obesity (50). The inverse relationship between HDL-C and bone turnover markers may reflect HDL dysfunction in obesity, rather than HDL-C levels per se. Obesity-related inflammation involves elevated cytokines (e.g., TNF-α, IL-6) that promote bone resorption. Dysfunctional HDL-C may lose its anti-inflammatory capacity, failing to counteract this process. Altered lipid metabolism in obesity also promotes bone marrow adipose tissue (BMAT) accumulation, which inversely correlates with bone mass. Impaired HDL-mediated reverse cholesterol transport may reduce cholesterol efflux from bone cells, favoring adipogenic over osteogenic differentiation and suppressing overall bone turnover, consistent with observed reductions in P1NP and β-CTX. The P1NP-to-β-CTX ratio acts as a measure of the equilibrium between bone creation and breakdown, with P1NP representing bone creation and β-CTX reflecting bone breakdown (33, 51). The strong correlation between β-CTX and P1NP illustrates the interplay between bone resorption and formation, as prior studies have identified P1NP and β-CTX as the most reliable indicators for forecasting changes in bone density (52). HDL may play a crucial role in maintaining bone health, potentially directly or indirectly regulating BTMs levels. In hospitalized patients with OPFs, HDL levels are linked to bone mass development, with reduced HDL levels exerting a protective effect on bone mass (38, 53). However, as HDL levels increase, the risk of bone loss in osteoporotic fracture hospitalized patients also increases. Specific oxysterols, as demonstrated in previous research, can stimulate mesenchymal stem cells’ differentiation into osteoblasts, while high HDL levels can remove oxysterols from peripheral tissues, negatively impacting osteoblastic differentiation (39).

In summary, these findings imply that reduced HDL levels may enhance bone density and prevent further progression of OP by lowering bone turnover, which is clinically relevant. Thus, according to the results of this study, healthcare providers should track and manage HDL levels in hospitalized OPFs patients to reduce bone resorption and improve bone density. This research screened OPFs hospitalized patients using precise statistical models and adjusted for key confounding factors. As far as we are aware, this represents the initial investigation carried out in China to examine the association between HDL levels and the markers P1NP and β-CTX in hospitalized osteoporosis-related fracture patients. Additionally, this research not only sheds light on the link between HDL and BMD but also proposes novel treatment approaches for patients in hospital settings.

This study has a number of limitations. Firstly, the retrospective cross-sectional nature of our study enables the detection of associations but does not allow for the establishment of causal links. This limitation is especially relevant because fracture incidents can trigger immediate physiological reactions that may change biomarkers that were previously stable. As a result, the detected relationships between HDL levels and BTMs may not completely represent causal mechanisms. Moreover, our study’s findings are based on a relatively modest sample size of 712 participants, which may not sufficiently represent the broader patient population with varied underlying conditions. Further studies with larger and more diverse cohorts are essential to validate and expand upon our findings.

Moreover, because our study was limited to hospitalized individuals with osteoporotic fractures, the applicability of these findings to the broader population is still uncertain. This specificity may limit the applicability of the conclusions outside the clinical setting of acute care for osteoporotic fractures. Finally, although our findings indicate a possible negative correlation between HDL levels and BTMs, suggesting that variations in lipid metabolism may affect bone turnover, the presence of unmeasured confounding variables could impact these relationships. Therefore, further research is necessary to confirm these preliminary observations and to elucidate the underlying biological mechanisms that link HDL with bone metabolism. Future studies should aim to include a broader range of confounding factors to improve the reliability and applicability of the results.

## 5 Conclusion

In our hospital, our research investigated the link between HDL levels and BTMs—specifically P1NP and β-CTX—in individuals with osteoporotic fractures. The results revealed an inverse relationship between HDL levels and both P1NP and β-CTX, implying that increased HDL levels could lead to reduced bone turnover. However, caution is needed in interpreting these findings due to the intricate effects of genetic, lifestyle, and clinical factors on lipid metabolism and bone turnover, which were not comprehensively explored in our study.

The study adds to current research by indicating a possible connection between lipid profiles and bone health, although these findings are preliminary. Further longitudinal and interventional studies are necessary to validate these findings and investigate the mechanisms underlying the relationship between HDL and bone turnover. This research may help establish whether modifying lipid profiles could be an effective approach for managing osteoporosis, potentially leading to the development of novel therapeutic strategies for the condition.

## Data Availability

The original contributions presented in the study are included in the article/supplementary material. Further inquiries can be directed to the corresponding author.
